# Multi-Scale Transient Thermo-Mechanical Coupling Analysis Method for the SiC_f_/SiC Composite Guide Vane

**DOI:** 10.3390/ma18143348

**Published:** 2025-07-17

**Authors:** Min Li, Xue Chen, Yu Deng, Wenjun Wang, Jian Li, Evance Obara, Zhilin Han, Chuyang Luo

**Affiliations:** 1 Hunan Aviation Powerplant Research Institute, Aero Engine Corporation of China, Zhuzhou 412000, China; limin0093@163.com (M.L.); mifeng_2005@163.com (W.W.); lijiannpu@163.com (J.L.); 2 Center for Civil Aviation Composites, Donghua University, Shanghai 201620, China; chenxuejh@163.com (X.C.); 2240014@mail.dhu.edu.cn (Y.D.); 424015@mail.dhu.edu.cn (E.O.); 3 College of Physics, Donghua University, Shanghai 201620, China; hanzhilin@dhu.edu.cn

**Keywords:** ceramic matrix composites, guide vane, multi-scale, thermal stress analysis, thermo-mechanical coupling

## Abstract

In composites, fiber–matrix thermal mismatch induces stress heterogeneity that is beyond the resolution of macroscopic approaches. The asymptotic expansion homogenization method is used to create a multi-scale thermo-mechanical coupling model that predicts the elastic modulus, thermal expansion coefficients, and thermal conductivity of ceramic matrix composites at both the macro- and micro-scales. These predictions are verified to be accurate with a maximum relative error of 9.7% between the measured and predicted values. The multi-scale analysis method is then used to guide the vane’s thermal stress analysis, and a macro–meso–micro multi-scale model is created. The thermal stress distribution and stress magnitudes of the guide vane under a transient high-temperature load are investigated. The results indicate that the temperature and thermal stress distributions of the guide vane under the homogenization and lamination theory models are rather comparable, and the locations of the maximum thermal stress are predicted to be reasonably close to one another. The homogenization model allows for the rapid and accurate prediction of the guide vane’s thermal stress distribution. When compared to the macro-scale stress values, the meso-scale predicted stress levels exhibit excellent accuracy, with an inaccuracy of 11.7%. Micro-scale studies reveal significant stress concentrations at the fiber–matrix interface, which is essential for the macro-scale fatigue and fracture behavior of the guide vane.

## 1. Introduction

Ceramic matrix composites (CMCs) are ideal for ultra-high-temperature applications because of their remarkable oxidation resistance, low density, high strength, and high temperature. They enable a 50% to 70% weight reduction in turbine engines, enhancing the thrust-to-weight ratio. Therefore, CMCs have emerged as an ideal candidate for turbine guide vanes and other hot-section components in next-generation aerospace engines [1,2]. However, high-temperature gas erosion and film cooling expose guide vanes to harsh transient temperature gradients during operation, resulting in high thermal stresses and failure, like interfacial debonding, matrix cracking, and oxidative damage that pose a serious risk to operational safety [3]. Therefore, it is essential to investigate how thermal stress is distributed in the CMC during transient high temperatures to guarantee the safety of engine operations.

The thermal response properties of CMC guide vanes under constant high-temperature settings have been the focus of extensive experimental investigation. Through thermal fatigue cycling experiments, Huo et al. [4] found that the CMC guide vane showed no notable damage features, such as internal or macroscopic cracks, when subjected to alternating high-temperature (1200 °C) and cooling (50 °C) conditions. The interfacial deterioration and matrix cracking properties of SiC_f_/SiC-CMC under cyclic loading at ambient temperature and 800 °C are demonstrated in [5]. Further studies indicated that the primary cause of high-temperature fatigue failure in SiC_f_/SiC-CMC is high-temperature oxidation damage [6]. Specifically, the oxidation of the fiber–matrix interface at 1100 °C leads to matrix crack propagation, significantly affecting the lifespan of SiC_f_/SiC-CMC [7]. The failure mode is characterized by the initiation of interfacial microcracks and the extension of cracks driven by oxidation under high-temperature cyclic loading [8]. To investigate the cyclic thermal shock behavior of braided SiC_f_/SiC-CMC tubes, Xu et al. [9] found that as the number of shock cycles increases, the fracture failure mode progressively changes from brittle to ductile. This is mostly because of the accumulation of thermal damage brought on by high-temperature strains inside the fiber bundles, which results in the development of microcracks and a notable reduction in tensile strength. However, experimental investigations find it difficult to capture the microscopic stress distributions and concentrations within the structure, even while they can directly acquire the macroscopic failure modes of composite materials under high-temperature thermal stresses. In recent years, the multi-scale analysis method has become a vital tool for examining the periodic structures of composite materials. Micro–meso–macro multilevel models have been developed by introducing a representative volume element (RVE) [10], which allows for accurate predictions of effective thermoelastic properties and stress–strain analysis of composite materials [11]. Sun et al. [12] calculated thermal expansion coefficients for C_f_/SiC composites between 100 °C and 1200 °C by applying a multi-scale model based on periodic displacement and temperature boundary conditions. Liang et al. [13] used micro-mechanical methods to present a tensile strength model for SiC_f_/SiC-CMC throughout a temperature range of 200 K to 2000 K. Furthermore, studies have investigated the effect of internal void, fiber content, weaving features, and fiber–matrix interface characteristics on the distribution of thermal stresses, leading to optimal composite structure designs that lower thermal stress levels [14].

The turbine engine undergoes three distinct states: startup, operation, and shutdown during service. As a result, the turbine guide vanes heat up quickly, operate at high temperatures, and cool down quickly. Significant amounts of thermal stress are generated inside the CMC during long-term service, which could cause cracks to form or spread throughout the matrix [15,16]. Therefore, to ensure their operational safety, it is imperative to investigate the factors producing transient thermal shock damage in the CMC. The multi-scale stress transfer mechanisms during transient thermo-mechanical changes are not sufficiently understood in the research, which has mostly focused on the thermo-mechanical response of CMC structures under steady-state high-temperature settings. Short-term stress peaks, interfacial debonding, and fracture propagation inside the structure are possible outcomes of CMC structures’ more complex and inherently multi-scale thermal stress production mechanism under transient thermo-mechanical coupling [17]. Additionally, the presence of internal voids considerably alters the material’s thermal conduction and expansion behavior when calculating the multi-scale thermal stresses in guide vanes. The higher void content accelerates fatigue failure processes by aggravating temperature gradients and possibly causing microcrack propagation, which may result in damage behaviors including interfacial debonding and crack extension [18,19]. Liu et al. [20] and Dong et al. [21] developed the void–matrix equivalent models to reflect the influence of voids in the matrix on the thermal properties of composite materials. Xu et al. [22] constructed a micro-mechanical model that considers the real manufacturing-induced voids. The prediction results with various void volume fractions (from 1% to 15%) indicated that the thermal conductivity decreases with the increasing void volume fraction. Sun et al. [23] established an RVE model to consider the effect of void content on the thermal conductivity of C/SiC composites and found that porosity has a significant impact on both in-plane and through-plane thermal conductivity. Therefore, it is necessary to consider the effect of matrix void content when conducting multi-scale thermal stress analysis. This study addresses this through analyzing thermal stresses in SiC_f_/SiC-CMC guiding vanes under transient high-temperature settings by applying a multi-scale transient thermo-mechanical coupling method. First, the RVE model of the CMC is established, and the voided SiC matrix’s thermoelastic properties are revised. The macro-scale and micro-scale effective thermoelastic properties are predicted and confirmed using the asymptotic expansion homogenization method (AEH). The thermal stress distributions in guide vanes under transient high-temperature conditions can then be quickly and accurately predicted due to the application of this multi-scale analytical method to the thermal stress analysis of guide vanes. This gives the structural design and operational safety evaluation of CMC guiding vanes a theoretical underpinning and methodological backing.

## 2. Multi-Scale Analysis Theory

### 2.1. Asymptotic Expansion Homogenization

The microscopic displacement vector for periodic composite materials depends on both microscopic coordinates (y) and macroscopic coordinates (x). Let y=x/k and define a small parameter k (0 < k ≤ 1) so that it is the ratio of the actual lengths of unit vectors in the microscopic coordinate system to those in the macroscopic scale. Let Φ be a function related to the composite material’s physical properties (e.g., displacement, stress, and temperature). Then, Φ shows periodicity with a period of *Y* and is a function of both macroscopic coordinates *x* and microscopic coordinates *y*. Any field function can be expressed in the following generic form [24]:

(1) $$\begin{array}{c} \Phi \left(x\right)=\Phi \left(x,y\right)=\Phi \left(x,y+Y\right) \end{array}$$

The displacement field $u_{i}$ can be asymptotically expanded in terms of the small parameter k as follows:

(2) $$\begin{array}{c} u_{i}=u_{i}^{k}\left(x\right)=u_{i}\left(x,y\right)=u_{i}^{\left(0\right)}\left(x,y\right)+ku_{i}^{\left(1\right)}\left(x,y\right)+k^{2}u_{i}^{\left(2\right)}\left(x,y\right)+\cdots \end{array}$$

The temperature field $T_{i}$ is expanded as follows:

(3) $$\begin{array}{c} T_{i}=T_{i}^{k}\left(x\right)=T_{i}\left(x,y\right)=T_{i}^{\left(0\right)}\left(x,y\right)+kT_{i}^{\left(1\right)}\left(x,y\right)+k^{2}T_{i}^{\left(2\right)}\left(x,y\right)+\cdots \end{array}$$

### 2.2. Homogenizations of Thermo-Mechanical Properties

The following is the process for predicting the homogenized parameter based on the previously given theory:

(1) Effective tensile modulus.

The stiffness tensors in different directions ($D_{ijkl}$) are computed under the AEH and a Python–Fortran homogenization plugin [25] using ABAQUS™, 2022 version. The following formula is used to determine Poisson’s ratios for anisotropic materials in various directions:

(4) $$\begin{array}{c} v_{xy}=-\frac{\varepsilon_{y}}{\varepsilon_{x}} \end{array}$$

where $v_{xy}$ represents the Poisson’s ratio in different directions, and $v_{23}=v_{32}$, $v_{12}=v_{13}$, $v_{21}=v_{31}$; Directions 1, 2, and 3 correspond to the fiber axial, radial, and thickness directions in the local coordinate system, respectively.

In orthotropic materials, the stress $\sigma_{xy}$ and strain $\varepsilon_{xy}$ relationship is given by Equation (5), in which $\gamma_{12}$, $\gamma_{13}$, and $\gamma_{23}$ are the shear strains in different directions.

(5) $$\begin{array}{c} \left\{\begin{array}{c} \begin{array}{c} \sigma_{11}\\ \sigma_{22} \end{array}\\ \begin{array}{c} \sigma_{33}\\ \sigma_{12} \end{array}\\ \begin{array}{c} \sigma_{13}\\ \sigma_{23} \end{array} \end{array}\right\}=\left[\begin{array}{cccccc} D_{1111} & D_{1122} & D_{1133} & 0 & 0 & 0\\ & D_{2222} & D_{2233} & 0 & 0 & 0\\ & & D_{3333} & 0 & 0 & 0\\ & & & D_{1212} & 0 & 0\\ & & & & D_{1313} & 1\\ & & & & & D_{2323} \end{array}\right]\left\{\begin{array}{c} \begin{array}{c} \varepsilon_{11}\\ \varepsilon_{22} \end{array}\\ \begin{array}{c} \varepsilon_{33}\\ \gamma_{12} \end{array}\\ \begin{array}{c} \gamma_{13}\\ \gamma_{23} \end{array} \end{array}\right\} \end{array}$$

The following Equation (6) provides the relationship between the stiffness tensors in different orientations and the tensile modulus, shear modulus, and shear strain:

(6) $$\begin{array}{c} \left\{\begin{array}{c} D_{1111}=E_{1}\left(1-v_{23}v_{32}\right)\gamma \\ \begin{array}{c} D_{2222}=E_{2}\left(1-v_{13}v_{31}\right)\gamma \\ \begin{array}{c} D_{3333}=E_{3}\left(1-v_{12}v_{21}\right)\gamma \\ \begin{array}{c} D_{1122}=E_{1}\left(v_{21}+v_{31}v_{23}\right)\gamma =E_{2}\left(v_{12}+v_{32}v_{13}\right)\gamma \\ \begin{array}{c} D_{1133}=E_{1}\left(v_{31}+v_{21}v_{32}\right)\gamma =E_{3}\left(v_{13}+v_{12}v_{23}\right)\gamma \\ D_{2233}=E_{2}\left(v_{32}+v_{12}v_{31}\right)\gamma =E_{3}\left(v_{23}+v_{21}v_{13}\right)\gamma \end{array} \end{array} \end{array} \end{array} \end{array}\right. \end{array}$$

where $E_{1}$, $E_{2}$, and $E_{3}$ are the tensile modulus values in the fiber, radial, and thickness directions, respectively; $G_{12}$, $G_{13}$, and $G_{23}$ are the shear modulus values in different directions; and γ is the overall shear strain, which is calculated using Equation (7).

(7) $$\begin{array}{c} \gamma =\frac{1}{1-v_{12}v_{21}-v_{23}v_{32}-v_{13}v_{31}-2v_{21}v_{32}v_{13}} \end{array}$$

(2) Effective thermal conductivity.

The initial temperature of each face of the RVE model is set to 25 °C to use the transient heat transfer approach to predict the effective thermal conductivity of CMC. A fixed heat flux density $q_{j}$ is then applied along each axial direction. After the temperature values of each axial element in the RVE model have been extracted, the heat conductivity in each direction is determined using Equation (8):

(8) $$\begin{array}{c} q_{j}=-k_{j}\frac{\Delta T}{A} \end{array}$$

where $q_{j}$ is the heat flux; $k_{j}$ is the thermal conductivity of the material in the *j* direction; ΔT is the temperature difference across the element in the *j* direction; and A is the cross-sectional area.

(3) Effective thermal expansion coefficients.

The following Equation (9) is used to compute the effective thermal expansion coefficients, which are obtained by extracting the thermal expansion displacements along each axis during temperature changes:

(9) $$\begin{array}{c} \alpha =\frac{1}{L}\frac{dL}{dT} \end{array}$$

where α is the thermal expansion coefficient; L is the original length of the sample, and $\frac{dL}{dT}$ is the relative elongation of the specimen when the temperature rises.

## 3. Multi-Scale Analysis and Result Validation

### 3.1. Multi-Scale Analysis Model

**Figure 1:**
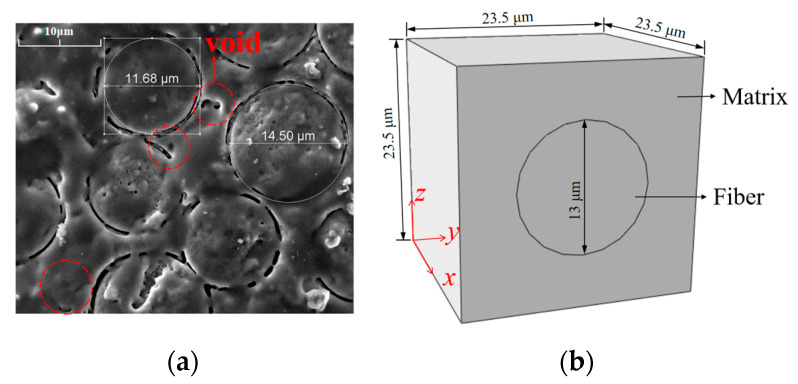
CMC micro-structure and RVE model. (**a**) Micro-structure. (**b**) RVE model.

Figure 1a displays the micro-structure of the SiC_f_/SiC-CMC as determined by scanning electron microscopy, with volume fractions of the fibers and matrix of $V_{f}$ = 24% and $V_{m}$ = 76%, respectively. Figure 1b displays the micro-scale RVE model of the SiC_f_/SiC-CMC, comprising cylindrical SiC fibers with a diameter of 13 μm and the SiC matrix with a side length of 23.5 μm. The coupled thermal stress analysis is performed in ABAQUS, where DC3D8 eight-node linear heat transfer hexahedron elements are used for discretization. The local coordinate system is established along the fiber axial, radial, and thickness directions and marked as x, y, and z.

In order to precisely describe the effect of the void content in the matrix, the volume percentage of voids ($V_{v}\prime$) must first be determined. Considering the fibers’ internal voids are typically believed to have a high-density structure, they are not considered here. The volume percentage of the non-voided matrix in the CMC is found to be $V_{m}\prime$ = 68.9% using Equation (10):

(10) $$\begin{array}{c} {\rho_{c}=\rho }_{SiC}(V_{f}+V_{m}\prime ) \end{array}$$

where $V_{f}$, $V_{m}\prime$, and $V_{m}$ represent the volume fractions of the SiC fiber, non-voided SiC matrix, and voided SiC matrix in SiC_f_/SiC-CMC, respectively; $\rho_{c}$ is the measured density of SiC_f_/SiC-CMC, which is 2.86 g/cm^3^; and $\rho_{SiC}$ is the theoretical density of SiC, which is 3.08 g/cm^3^.

Then, using Equation (11), the void fraction in the CMC is calculated as $V_{v}$ = 7.1%, leading to the void fraction within the matrix, $V_{v}\prime$ = $V_{v}$/$V_{m}$ = 7.1%/76% = 9.34%:

(11) $$\begin{array}{c} V_{f}+V_{m}\prime +V_{v}=1 \end{array}$$

where $V_{v}$ represents the volume fractions of the voids in SiC_f_/SiC-CMC.

**Figure 2:**
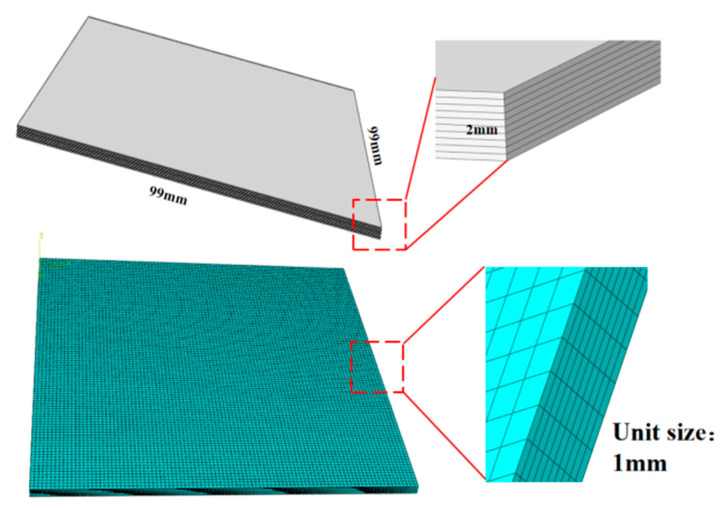
Finite element model of macroscopic laminated plates.

By the interfacial continuity, we established a macro-scale laminated model of SiC_f_/SiC-CMC that has dimensions of 99 mm × 99 mm × 2 mm and a thickness of 0.2 mm for each layer. As shown in Figure 2, DC3D8 eight-node linear heat transfer hexahedron elements with a mesh size of 1 mm are used to discretize the laminate, which is handled as a continuous and uniform entity. Seed points are strategically defined along the geometry and the boundaries to adjust the local refinement.

### 3.2. Revision of Matrix Thermoelastic Properties

The 0° tensile modulus, 0° and 90° thermal conductivities, 0° and 90° thermal expansion coefficients, specific heat capacity, and density at room temperature of SiC_f_/SiC-CMC were measured using standards GJB 8736-2015 [26], GB/T 22588-2008 [27], GB/T 16535-2008 [28], and GB/T 25995-2010 [29] as the benchmark for subsequent CMC parameter prediction to verify the accuracy of the predicted thermoelastic properties of SiC_f_/SiC-CMC through component thermoelastic properties in multi-scale analysis. The thermoelastic properties of SiC fibers were provided by Fujian Leadasia New Material Co., Ltd. in China The thermoelastic properties of the SiC matrix were extracted from the literature, and the measured thermoelastic properties of the CMC are listed in Table 1. Nevertheless, the literature usually ignores the effect of voids within the matrix by assuming that the SiC matrix is either non-voided or has a low void content. As a result, in this study, the thermoelastic properties of the SiC matrix are revised by introducing a void content, $V_{v}\prime$ = 9.34%.

In the revision of the thermal expansion coefficient and specific heat capacity, given that the void volume percentage is low and the expansion behavior of the gas within the voids is relatively independent of the atomic structure of the matrix phase, and considering that the material’s heat capacity is predominantly governed by the chemical bonds and thermal vibrations between atoms in the matrix, the coefficient of thermal expansion and heat capacity should not vary with void content. Thus, the literature extraction values for the SiC matrix are directly adopted as the revised thermal expansion coefficient and specific heat capacity.

The following are the revised methods for calculating thermal conductivity, density, and tensile modulus: For thermal conductivity, the I-Maxwell inverse effective thermal conductivity model [34] is utilized to establish the relationship between the thermal conductivity of the voided matrix and that of the non-voided matrix, as expressed in Equation (12). The revised thermal conductivity of the voided matrix is obtained by entering the non-voided matrix’s thermal conductivity from Table 1 into Equation (12):

(12) $$\begin{array}{c} k_{m,r}=k_{m}\prime \frac{2k_{m}\prime +k_{p}+2V_{v}\prime \left(k_{p}-k_{m}\prime \right)}{2k_{m}\prime +k_{p}-V_{v}\prime \left(k_{p}-k_{m}\prime \right)} \end{array}$$

where $k_{m,r}$ is the revised thermal conductivity of the voided matrix; $k_{m}\prime$ and $k_{p}$ are the thermal conductivities of the matrix phase and the void phase (air) in the voided SiC matrix, respectively, with values of $k_{m}\prime$ = 90 W/(m·K) and $k_{p}$ = 0.026 W/(m·K).

The density revision method for voided materials is shown in Equation (13):

(13) $$\begin{array}{c} \rho_{m,r}=\rho_{m}\prime V_{m}\prime +\rho_{v}\prime V_{v}\prime \end{array}$$

where $\rho_{m,r}$ is the revised density of the voided matrix; $\rho_{m}\prime$ and $\rho_{v}\prime$ are the densities of the matrix phase and the void phase (air) in the voided matrix material, respectively, with values of $\rho_{m}\prime$ = 3.08 g/cm^3^ and $\rho_{v}\prime$ = 1.29 × 10^−3^ g/cm^3^.

For materials with higher void content and complex void structures, nonlinear models such as exponential or polynomial formulations can provide higher fidelity in property prediction [35,36]. In contrast, due to the relatively low void content (9.34%) and the assumption of isotropic void distribution in this study, the tensile modulus of the voided matrix is revised for mechanical properties using the rule of mixtures, which is based on weighted averages [37]. Equation (14) provides the link between the tensile modulus of the non-voided matrix and that of the voided matrix:

(14) $$\begin{array}{c} E_{m,r}=E_{m}\prime V_{m}+E_{v}V_{v} \end{array}$$

where $E_{m,r}$ is the revised tensile modulus of the voided matrix; $E_{m}\prime$ and $E_{v}$ are the tensile modulus values of the matrix phase and the void phase (air) in the voided matrix material, respectively, with values of $E_{m}\prime$ = 350 GPa and $E_{v}$ = 0.

In conclusion, Table 2 shows the thermoelastic properties of the voided SiC matrix (referred to as the “Revised SiC Matrix”) using the previously discussed revised methodologies. Along with the measured properties of SiC_f_/SiC-CMC and the SiC fiber properties from Table 1, the theoretical thermoelastic properties of the real voided SiC matrix are inversely calculated using micro-mechanics theory to determine the accuracy of the aforementioned revised methodologies. The accuracy of the revisions is then confirmed by comparing these theoretical values with the revised properties using error analysis. First, Equation (15) expresses the inverse computation for the tensile modulus:

(15) $$\begin{array}{c} E_{m,t}=\frac{E_{c}{-E}_{f}V_{f}}{V_{m}} \end{array}$$

where $E_{m,t}$ is the theoretical modulus of the voided matrix; $E_{c}$ and $E_{f}$ are the measured tensile modulus values of SiC_f_/SiC-CMC at 0° and the tensile modulus of the fiber at 0°, respectively, with values of $E_{c}$ = 319.17 GPa and $E_{f}$ = 370 GPa.

The inversion formula for the density of the voided matrix is as follows:

(16) $$\begin{array}{c} \rho_{m,t}=\frac{\rho_{c}-\rho_{f}V_{f}}{V_{m}} \end{array}$$

where $\rho_{m,t}$ is the theoretical density of the voided matrix; $\rho_{f}$ is the density of the SiC fiber, with a value of 3.08 g/cm^3^.

The inversion formula for thermal conductivity is shown in Equation (17) as follows:

(17) $$\begin{array}{c} k_{m,t}=\frac{k_{c}-V_{f}k_{f}}{V_{m}} \end{array}$$

where $k_{m,t}$ is the theoretical thermal conductivity of the voided matrix; $k_{c}$ and $k_{f}$ are the measured thermal conductivity of the CMC and the fiber in the 0° direction, with values of $k_{c}$ = 65.68 W/(m·K) and $k_{f}$ = 14.7 W/(m·K).

The inversion formula for specific heat capacity is shown in Equation (18) as follows:

(18) $$\begin{array}{c} C_{m,t}=\frac{C_{c}-C_{f}V_{f}}{V_{m}} \end{array}$$

where $C_{m,t}$ is the theoretical specific heat capacity of the voided matrix; $C_{c}$ and $C_{f}$ are the measured specific heat capacities of the CMC and the fiber, with values of $C_{c}$ = 0.65 J/(g·K) and $C_{f}$ = 0.71 J/(g·K).

The inversion formula for the thermal expansion coefficients is shown in Equation (19) as follows:

(19) $$\begin{array}{c} \alpha_{m,t}=\frac{\alpha_{c}\left(V_{f}E_{f}+V_{m}E_{m,t}\right)-\alpha_{f}V_{f}E_{f}}{V_{m}E_{m,t}} \end{array}$$

where $\alpha_{m,t}$ is the theoretical thermal expansion coefficient of the voided matrix; $\alpha_{c}$ and $\alpha_{f}$ are the measured thermal expansion coefficients of the CMC in the 0° direction and the fiber in the 0° direction, with values of $\alpha_{c}$ = 5.20 × 10^−6^/K and $\alpha_{f}$ = 3.76 × 10^−6^/K.

Table 2 presents the theoretically thermoelastic properties of the voided SiC matrix (referred to as the “Theoretical SiC Matrix”). Using Equation (20), the error between the revised values and the theoretical values, represented as Error 1, is calculated. The error values are found to be less than 10%, underscoring the high accuracy of the revised values and their suitability for multi-scale thermoelastic analysis.

(20) $$\begin{array}{c} Error\;1=\frac{revised\;value-theoretical\;value}{theoretical\;value} \end{array}$$

### 3.3. Predictions and Validations of Effective Thermoelastic Properties

#### 3.3.1. Predictions and Validations of Micro-Scale Effective Thermoelastic Properties

The SiC fiber properties from Table 1 and the Revised SiC Matrix properties from Table 2 are used to set the material properties for the micro-scale RVE model. Employing the AHE and the RVE model, the stiffness components of the micro-scale RVE model in various directions are first calculated using a homogenization plugin. To calculate the Poisson’s ratio of the unit cell, one face of the RVE model is fixed along the x, y, and z axes, and the other face is subjected to a tensile load of 10 N/m^2^. This allows for the measurement of axial positive strain and transverse negative strain in various directions. Ultimately, Table 3 displays the stiffness components and Poisson’s ratio results for the orthotropic anisotropic material in various directions. As seen in Table 4, the effective modulus is computed using the anisotropic material’s stress–strain relationship. The tensile modulus shows a relative error of 3.7% when compared to measured values, as determined by applying Equation (21). The error in this study is significantly lower than the tensile modulus predictions of the RVE model in Reference [38], which also used FEM combined with asymptotic homogenization to predict the thermo-mechanical behavior of 2D SiC_f_/SiC composites, but did not take void into account and reported an error of 11%. This reduction emphasizes how crucial it is to include void-revised matrix properties to improve the tensile modulus of CMC prediction precision. The mechanical response of CMC can be effectively predicted at the micro-scale by revising the matrix tensile modulus for void effects.

(21) $$\begin{array}{c} Error\;2=\frac{measured\;value-predicted\;value}{measured\;value} \end{array}$$

**Figure 3:**
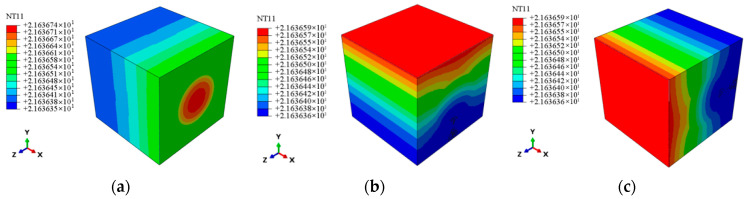
The nephogram of axial temperature distribution at the meso-scale. (**a**) *x* axis. (**b**) *y* axis. (**c**) *z* axis.

A heat flux load of 1 W/m^2^ is applied to each face of the RVE model to predict effective thermal conductivity. This causes the temperature field distributions as seen in Figure 3. The effective thermal conductivity in each axial direction is determined by taking the element temperature values along each axis and applying Equation (11); the effective thermal conductivity is reported in Table 5. Error analysis is performed by comparing these results with the measured thermal conductivities in the fiber 0° direction and the thickness direction, yielding relative errors of 8.3%, 6.7%, and 6.7%, respectively. Reference [39] showed a prediction error of 20.29% for thermal conductivity, taking into account the role of voids in CMC and revising the SiC matrix’s effective properties appropriately. In contrast, this study greatly increased the prediction accuracy of thermal conductivity at the micro-scale by employing revised methods to determine the thermal conductivity of the voided SiC matrix.

**Figure 4:**
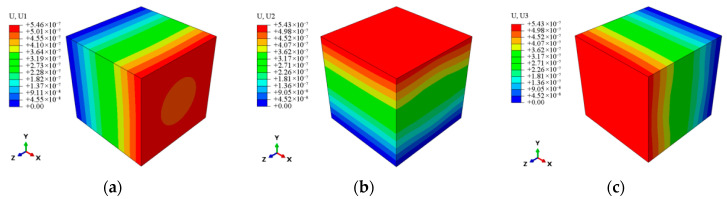
The nephogram of axial displacement distribution at the meso-scale. (**a**) *x* axis. (**b**) *y* axis. (**c**) *z* axis.

In the prediction of the effective thermal expansion coefficients, all elements in the RVE model have their initial temperature set to 20 °C. Then, one axial face’s temperature is raised to 25 °C, which is opposite to the vertical axial face, and subjected to corresponding axial constraints to ensure fixed boundary conditions and directional thermal expansion. Figure 4 shows the displacement variations along each axis during temperature change. By calculating the relative axial elongation of the specimen in the RVE model and using Equation (12), the axial expansion displacements for a temperature increase of 5 °C are determined, yielding the effective thermal expansion coefficients in different directions, as presented in Table 6. The relative errors of 8.6%, 1.5%, and 1.5% in each of the three directions when compared to the measured thermal expansion coefficients demonstrate that the thermal expansion coefficients that are predicted can be used to analyze the thermoelastic responses of CMC guide vanes at the multi-scale.

#### 3.3.2. Predictions and Validations of Macro-Scale Effective Thermoelastic Properties

**Figure 5:**
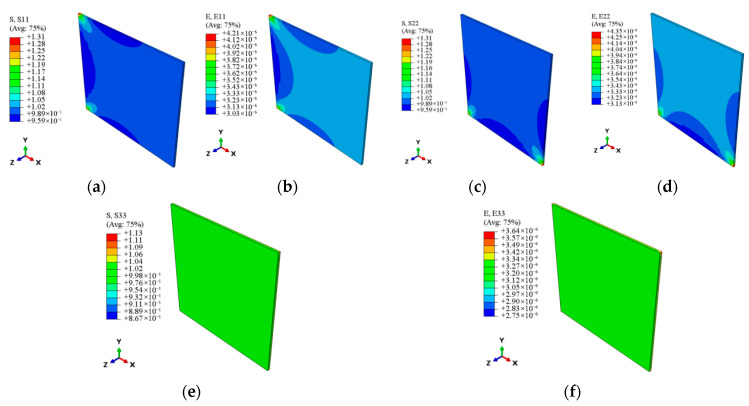
The nephogram of axial stress–strain distribution at the macro-scale. (**a**) *x* axis stress. (**b**) *x* axis strain. (**c**) *y* axis stress. (**d**) *y* axis strain. (**e**) *z* axis stress. (**f**) *z* axis strain.

**Figure 6:**
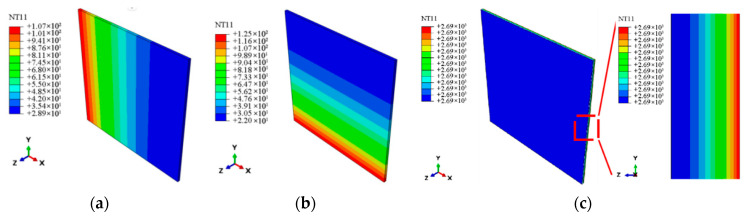
The nephogram of axial temperature distribution at the macro-scale. (**a**) *x* axis temperature. (**b**) *y* axis temperature. (**c**) *z* axis temperature.

**Figure 7:**
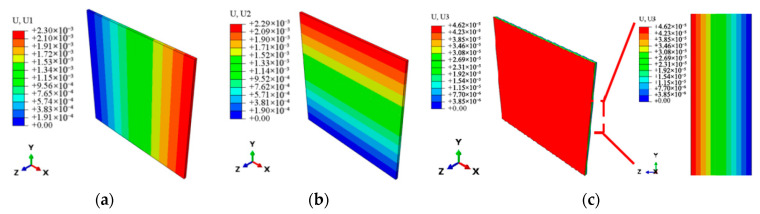
The nephogram of axial displacement distribution at the macro-scale. (**a**) *x* axis displacement. (**b**) *y* axis displacement. (**c**) *z* axis displacement.

The effective thermoelastic properties at the micro-scale are homogenized into overall effective properties using the AEH and transferred to the macro-scale laminate model. The same loading conditions and boundary constraints as those in Section 3.3.1 are applied to verify the cross-scale effectiveness. Figure 5, Figure 6 and Figure 7 show the stress, strain, temperature, and expansion displacement of the macro-scale laminate model, respectively. The effective tensile modulus, thermal conductivities, and thermal expansion coefficients in various axial directions at the macro-scale are anticipated by extracting the element data along each model axis, as shown in Table 7. The findings show that the laminate model well depicts the material’s macroscopic thermoelastic behavior, with predicted effective thermoelastic properties showing errors of less than 10% when compared to the measured values.

## 4. Multi-Scale Analysis of Thermal Stress in the Guide Vane

### 4.1. Macro-Scale Thermal Stress Distribution and Homogenization Method Validation

**Figure 8:**
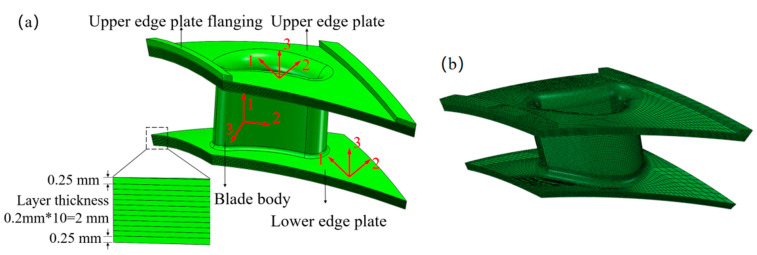
The finite element model of the guide vane. (**a**) Finite element model; (**b**) Grid division.

To assess the effectiveness of the homogenized method in the thermal stress analysis of the guide vane, a finite element model of the SiC_f_/SiC-CMC guide vane is initially established based on laminate theory, as shown in Figure 8. The vane body has a thickness of 2.5 mm and follows a layup arrangement of [0/90]_3S_, wherein six 0° layers are flanged upward and downward to form part of the upper and lower flange plates. Both the upper and lower flange plates possess a thickness of 2.5 mm, each adopting a layup scheme of [0/45/0/-45/0/0]_S_. The flange of the upper edge plate measures 3.0 mm in thickness, with a layup arrangement of [0/45/0/-45/0/0/-45/45/0/0/-45/0/45/0]. The coordinate systems for the vane body and the upper and lower flange plates are defined as depicted in Figure 8a, with 0° aligned along the axial direction of the vane body, 90° along its circumferential direction, and the 1, 2, and 3 axes corresponding to the 0°, 90°, and thickness directions, respectively. The laminate model’s thermoelastic characteristics are obtained from the CMC properties given in Table 1. When conducting heat conduction simulation, the macro-scale model is meshed with DC3D8 eight-node linear heat transfer hexahedron elements and 227,118 nodes, and seeding is set at 1.5 mm, while C3D8R eight-node hexahedral linear reduced integration elements are used for thermal stress simulation.

The laminate model is equated to a homogeneous material to create a homogenization model, with the homogenized material properties obtained through the asymptotic homogenization property prediction method, as shown in Table 8. For the heat conduction prediction, all model elements have an initial temperature of 20 °C, followed by a rapid temperature increase to 1200 °C at a rate of 20 °C/s, which is then maintained at 1200 °C for 60 s. The exterior surfaces that surround the vane body are subjected to the thermal load.

**Figure 9:**
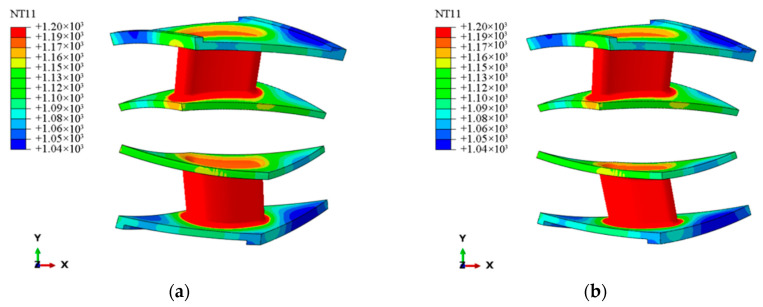

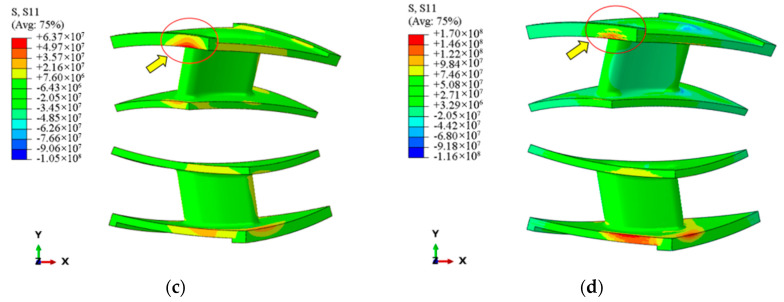
Temperature and thermal stress nephogram under homogenized and laminated models. (**a**) Homogenized structure temperature. (**b**) Laminated structure temperature. (**c**) Homogenized structure thermal stress. (**d**) Laminated structure thermal stress.

Figure 9a,b show the temperature distribution nephogram under the multi-scale homogenized model and the laminate model. It can be observed that the temperature distributions in both models are consistent, with the highest temperature distribution located in the vane body, reaching a maximum temperature of 1200 °C, and the lowest temperature distribution located at the flange of the upper edge plate, where the temperature is 1040 °C. These results demonstrate that the temperature field distribution inside the guide vane can be precisely captured by the multi-scale homogenization method, offering a reliable thermal load base for further thermal stress research. Figure 9c,d further presents the thermal stress distribution nephogram; both models indicate that the maximum thermal stress is concentrated in the upper flange plate (the part circled in red), with the macroscopic stress field distributions exhibiting similar trends. This suggests that the multi-scale homogenization method accurately predicts the locations of high thermal stress regions and captures the main stress characteristics arising from thermo-mechanical coupling effects. Although the homogenization model predicts lower thermal stress levels than the laminate model, especially in regions with localized stress concentration, the highest stress magnitude for the laminated model is 170 MPa, while the maximum stress magnitude for the homogenized model is 64 MPa, where the laminate model shows larger peak stresses. This discrepancy arises primarily from the presence of stress concentration regions near the geometric corners of the blade body. The laminated model is capable of more accurately capturing such stress concentration effects, whereas the homogenized model, due to its inherent assumption of material continuity, fails to reflect the localized stress concentrations induced by anisotropy. Moreover, the laminated structure can explicitly represent the anisotropic characteristics between individual layers, as well as the interlaminar thermal stress variations resulting from mismatches in the coefficients of thermal expansion among different constituent materials. The homogenization method simplifies the microscopic heterogeneity, smoothing out interlayer thermal expansion mismatches and interfacial stress concentration effects through equivalent continuum theory. This leads to a reduction in the apparent stress concentration, particularly near ply interfaces or discontinuities. The laminate model can reflect the thermal stress differences between different layers in detail. Overall, the multi-scale homogenization method proves effective in predicting the macroscopic thermal stress distribution and identifying high-stress regions in CMC guide vanes. Furthermore, the adoption of a properties revision approach that accounts for void effects streamlines the construction of voided models, offering significant advantages in the rapid evaluation of the thermo-mechanical response of complex structures.

### 4.2. Meso-Scale Thermal Stress Analysis

**Figure 10:**
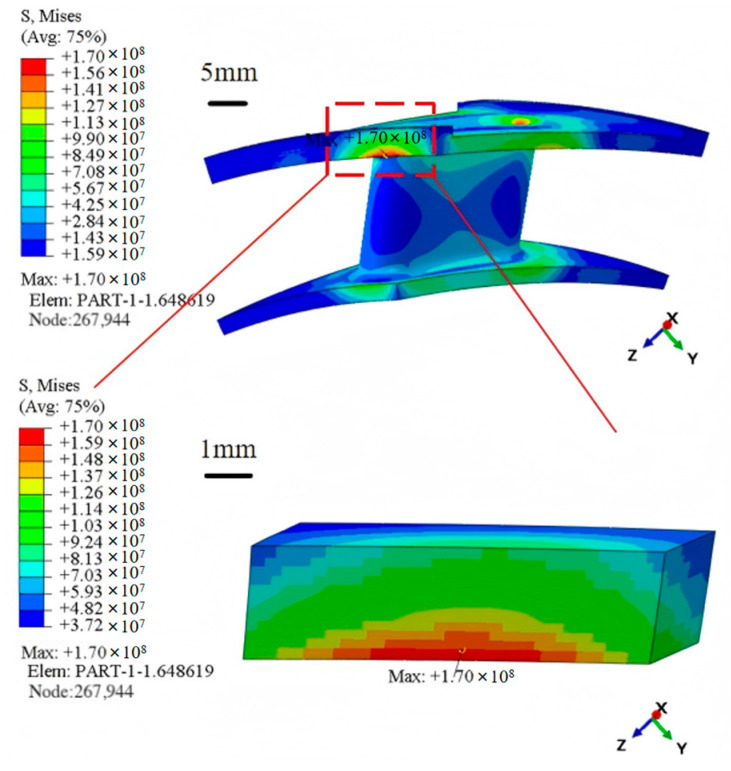
Extraction of maximum stress elements at the macro-scale.

**Figure 11:**
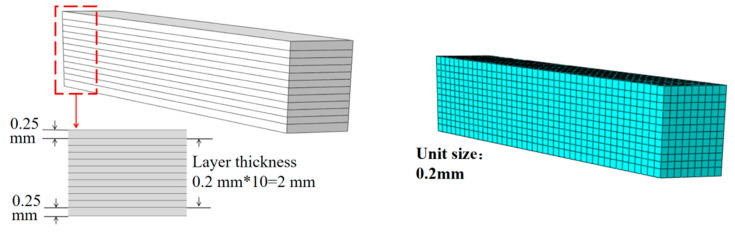
Finite element model at the meso-scale.

The unit with the highest stress within the homogenized model (shown in Figure 10) is selected for creating a meso-scale laminate model (shown in Figure 11) based on the findings of the macroscopic thermal stress distribution. The imposed temperature load matches the value acquired from the macro-scale computations in Section 4.1, and the mesh size is 0.2 mm. The meso-scale model is meshed with DC3D8 eight-node linear heat transfer hexahedron elements, 4536 nodes, and seeding is set at 0.2 mm. Discretization is carried out using DC3D8 linear hexahedral elements similar to the macro-scale to ensure compatibility of the thermal stress results and to maintain computational tractability.

**Figure 12:**
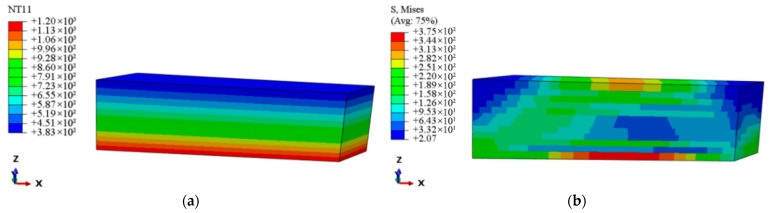
Temperature and thermal stress distribution at the meso-scale. (**a**) Temperature distribution. (**b**) Thermal stress distribution.

Figure 12 displays the temperature and thermal stress distribution nephogram at the meso-scale. The results indicate that the temperature at the meso-scale decreases progressively along the direction of heat transfer, aligning closely with the temperature distribution trend observed at the maximum stress element at the macro-scale. The stress determined from the meso-scale model study is approximately 189 MPa when the effects of stress concentration are taken out of the calculations. Compared to the maximum thermal stress of 170 MPa from the macro-scale model shown in Figure 9d, this yields an error of 11.7%. This result demonstrates how well the meso-scale laminate model predicts the magnitudes of thermal stress. The meso-scale model more accurately depicts the consequences of thermal expansion mismatch between layers by fine-tuning the interlayer structure of the composite turbine guide vane, resulting in improved accuracy in thermal stress predictions. In a similar study, Shen et al. [38] obtained an approximated 11% error between the predicted values and experimental measurements, further validating the accuracy of the current work.

### 4.3. Micro-Scale Thermal Stress Analysis

**Figure 13:**
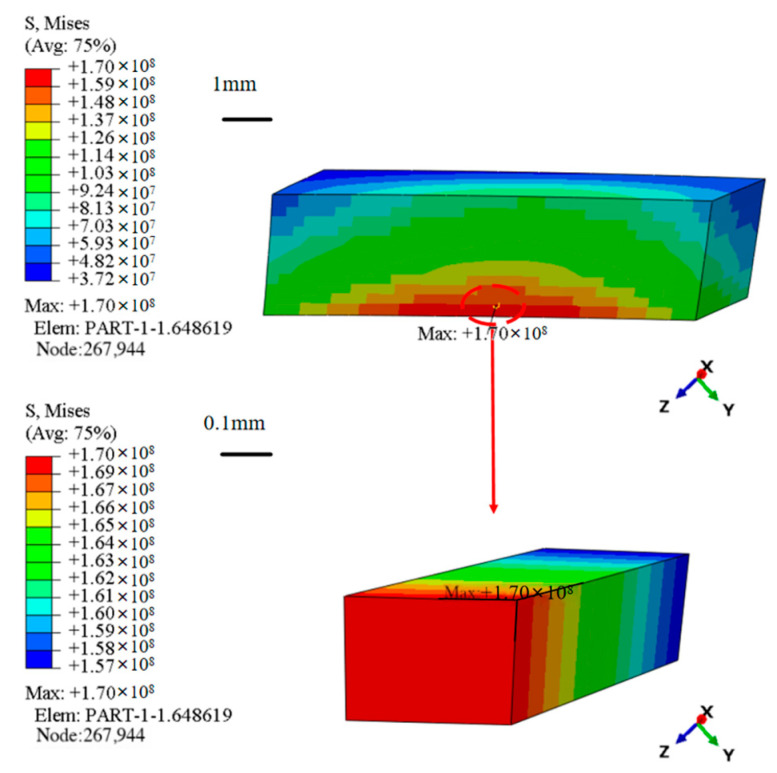
Extraction of maximum stress elements at the meso-scale.

**Figure 14:**
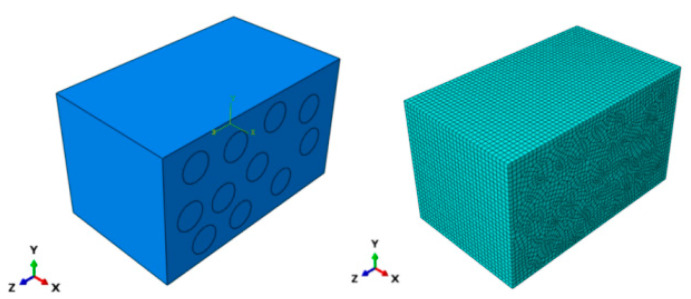
Finite element model at the micro-scale.

As shown in Figure 13, the region of maximum thermal stress within the meso-scale laminate model is extracted. Given that the fibers in this region are oriented along the 0° direction, a micro-scale RVE model is established with fibers aligned along the x-direction (see Figure 14). The micro-scale model is meshed with DC3D8 eight-node linear heat transfer hexahedron elements, 54,930 nodes, and seeding is set at 0.002 mm. The same temperature boundary conditions as those applied to the meso-scale model are imposed.

**Figure 15:**
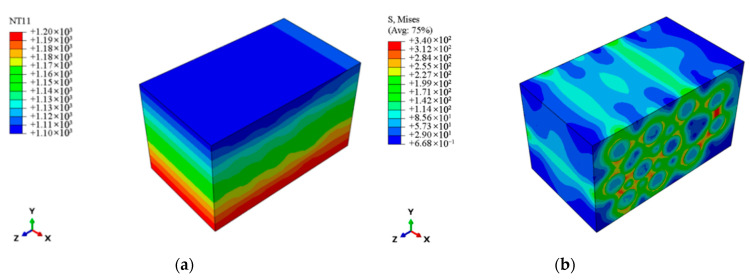
Temperature and thermal stress distribution at the micro-scale. (**a**) Temperature distribution. (**b**) Thermal stress distribution.

Figure 15a displays the temperature distribution nephogram at the micro-scale. The results reveal that, due to differences in thermal conductivity between the fiber and matrix, localized perturbations in heat flux density occur, leading to periodic temperature fluctuations in the interfacial region with a wavelength of approximately 5 μm and an amplitude of *ΔT* ≈ 50 K. Figure 15b presents the stress distribution at the micro-scale. The RVE theory and the Hill–Mandel macro–micro consistency principle [40] are employed to determine the average stress of the RVE model. The stress distribution throughout the entire micro-scale structure is integrated using a volume-weighted approach, yielding an effective average stress of 1.25 × 10^2^ MPa. When compared to the maximum thermal stress of 1.70 × 10^2^ MPa at the macro-scale of the guide vane, the error falls within an acceptable range, thus validating the accuracy of the cross-scale finite element method based on the thermoelastic performance properties of micro-scale constituents in predicting thermal stresses. Furthermore, the temperature gradient, resulting from the mismatch in thermal expansion coefficients between the fiber (α*_f_* = 3.76 × 10^−6^/K) and the matrix (α*_m_* = 4.7 × 10^−6^/K), induces thermal stress concentration at the interface. The local maximum thermal stress reaches 340 MPa, with the width of the concentration zone being approximately 0.8 times the fiber diameter. This result aligns with the findings in [41,42], where localized thermal stress peaks due to geometric and material discontinuities were shown to critically impact damage initiation, such as matrix microcrack propagation and degradation of interfacial bonding strength [43]. Then, a stress concentration factor ($k_{t}$) is introduced to quantitatively evaluate the local thermal stress amplification effect within the micro-scale structure, defined as the ratio of the local maximum stress $\sigma_{max}$ to the average stress $\sigma_{avg}$. The calculated $k_{t}$ value of 2.72 indicates the presence of a significant stress concentration region at the fiber–matrix interface. This region not only serves as a critical initiator of micro-scale damage but also exerts a substantial effect on the fatigue and fracture performance of the guide vane at the macro-scale.

## 5. Conclusions

A multi-scale transient thermo-mechanical coupling analysis method, based on a void-revised RVE model, was created for the SiC_f_/SiC-CMC guide vane. The accuracy of this method in predicting the effective thermoelastic properties, thermal stress distribution, and stress magnitudes of CMC guide vanes is substantiated through theoretical analysis and experimental validation. The following summarizes the main findings:

(1) A revision model based on micro-mechanics theory is established for the properties of the voided SiC matrix by combining the micro-scale RVE model with the asymptotic expansion homogenized method. This approach enabled precise cross-scale prediction of the effective thermoelastic properties of SiC_f_/SiC-CMC. The maximum relative error between the model predictions and measured validation is determined to be 9.7%, affirming the method’s effectiveness in characterizing the thermoelastic properties of SiC_f_/SiC-CMC.

(2) A hierarchical macro–meso–micro multi-scale model of the guide vane is created to analyze its thermal stress distribution and stress magnitude under transient high-temperature conditions. The findings indicate that the maximum stress is located at the upper flange plate of the guide vane. A high degree of accuracy in predicting the magnitude of stress is demonstrated by the meso-scale stress prediction, which showed an error of 11.7%. Micro-scale analysis further reveals a pronounced stress concentration region at the fiber–matrix interface, with a model-derived stress concentration factor of 2.72. This concentration significantly influences the fatigue and fracturing performance of the guide vane at the macro-scale.

## Figures and Tables

**Table 1:** Thermoelastic properties of SiC_f_/SiC-CMC, SiC fiber, and the SiC matrix.

Property	SiC_f_/SiC-CMC	SiC Fiber	SiC Matrix (Literature, Excluding Voids)
Tensile modulus E	319.17 GPa	370 GPa	350 GPa [30]
Density ρ	2.86 g/cm^3^	3.08 g/cm^3^	3.08 g/cm^3^
Thermal conductivity *k*	*λ*∥ = 65.68 W/(m·K) *λ*⊥ = 40.78 W/(m·K)	*λ*∥ = 14.7 W/(m·K) *λ*⊥ = 11.7 W/(m·K)	90 W/(m·K) [31]
Specific heat capacity *C*	0.65 J/(g·K)	0.71 J/(g·K)	0.68 J/(g·K) [32]
Thermal expansion coefficients *α*	*α*∥ = 5.20 × 10^−6^/K *α*⊥ = 4.55 × 10^−6^/K	*α*∥ = 3.76 × 10^−6^/K *α*⊥ = 4.15 × 10^−6^/K	4.7 × 10^−6^/K [33]

Note: ∥ represents the fiber 0° direction and ⊥ represents the fiber vertical direction.

**Table 2:** Error values of revised and thermoelastic properties of the SiC matrix.

Property	Revised SiC Matrix	Theoretical SiC Matrix (Including Voids)	Error 1
Tensile modulus *E*	317.31 GPa	303.12 GPa	4.68%
Density *ρ*	2.79 g/cm^3^	2.79 g/cm^3^	0%
Thermal conductivities *k*	78.03 W/(m·K)	81.77 W/(m·K)	4.61%
Specific heat capacity *C*	0.68 J/(g·K)	0.63 J/(g·K)	7.94%
Thermal expansion coefficients *α*	4.7 × 10^−6^/K	5.22 × 10^−6^/K	9.96%

**Table 3:** Stiffness component and Poisson’s ratio.

Stiffness Component (GPa)						
*D*_1111_	*D*_1122_		*D*_2222_	*D*_1133_		*D*_2233_
347.19	60.44		336.15	50.55		60.71
*D*_3333_	*D*_1212_		*D*_1313_	*D*_2323_		-
334.67	135.40		135.38	135.32		-
Poisson’s ratio						
*v*_12_		*v*_13_			*v*_23_	
0.147		0.152			0.149	

**Table 4:** Error values between the predicted and experimental tensile modulus.

Predicted Value (GPa)	Experimental Value (GPa)	Error 2
*E*_1_ = 307.42	$E_{1}$ = 319.17	3.7%
$E_{2}$ = 297.96	-	-
$E_{3}$ = 296.40	-	-
$G_{1}$ = 135.40	-	-
$G_{2}$ = 135.38	-	-
$G_{3}$ = 135.32	-	-

**Table 5:** Error values between the predicted and experimental thermal conductivities.

Predicted Value W/(m·K)	Experimental Value W/(m·K)	Error 2
$\lambda_{1}$ = 60.25	$\lambda_{1}$ = 65.68	8.3%
$\lambda_{2}$ = 43.51	$\lambda_{2}$ = 40.78	6.7%
$\lambda_{3}$ = 43.51	$\lambda_{3}$ = 40.78	6.7%

**Table 6:** Error values between the predicted and experimental thermal expansion coefficients.

Predicted Value	Experimental Value	Error 2
$\alpha_{1}$ = 4.75 × 10^−6^/K	$\alpha_{1}$ = 5.20 × 10^−6^/K	8.6%
$\alpha_{2}$ = 4.62 × 10^−6^/K	$\alpha_{2}$ = 4.55 × 10^−6^/K	1.5%
$\alpha_{3}$ = 4.62 × 10^−6^/K	$\alpha_{3}$ = 4.55 × 10^−6^/K	1.5%

**Table 7:** Error of thermoelastic properties at the macro-scale.

Thermoelastic Properties	*x* Axis	*y* Axis	*z* Axis
Effective tensile modulus (GPa)	307.42	297.69	296.40
Measured tensile modulus (GPa)	319.17	-	-
Error of tensile modulus	3.68%	-	-
Effective thermal conductivities (w/(m·k))	60.25	43.51	43.51
Measured thermal conductivities (w/(m·k))	65.68	40.78	40.78
Error of thermal conductivity	8.27%	6.69%	6.69%
Effective thermal expansion coefficients	4.75 × 10^−6^/K	4.62 × 10^−6^/K	4.62 × 10^−6^/K
Measured thermal expansion coefficients	5.20 × 10^−6^/K	4.55 × 10^−6^/K	4.55 × 10^−6^/K
Error of thermal expansion coefficients	8.65%	1.54%	1.54%

**Table 8:** Properties of the homogenized material.

The homogenized mechanical and thermal properties of the blade body					
*E*_1_	*E*_2_	*E*_3_	*G*_12_	*G*_13_	*G*_23_
302.6 GPa	302.6 GPa	296.40 GPa	135.40 GPa	135.38 GPa	135.32 GPa
$v_{12}$	$v_{13}$	$v_{23}$	$v_{21}$	$v_{31}$	$v_{32}$
0.144	0.149	0.144	0.152	0.152	0.149
$\lambda_{1}$		$\lambda_{2}$		$\lambda_{3}$	
51.88 W/(m·K)		51.88 W/(m·K)		43.51 W/(m·K)	
$\alpha_{1}$		$\alpha_{2}$		$\alpha_{3}$	
4.685 × 10^−6^/K		4.685 × 10^−6^/K		4.62 × 10^−6^/K	
The homogenized mechanical and thermal properties of the upper/lower edge plate					
*E*_1_	*E*_2_	*E*_3_	*G*_12_	*G*_13_	*G*_23_
303.7 GPa	303.7 GPa	296.40 GPa	135.36 GPa	135.36 GPa	135.32 GPa
$v_{12}$	$v_{13}$	$v_{23}$	$v_{21}$	$v_{31}$	$v_{32}$
0.149	0.147	0.149	0.152	0.152	0.149
$\lambda_{1}$		$\lambda_{2}$		$\lambda_{3}$	
49.8 W/(m·K)		49.8 W/(m·K)		43.51 W/(m·K)	
$\alpha_{1}$		$\alpha_{2}$		$\alpha_{3}$	
4.69 × 10^−6^/K		4.69 × 10^−6^/K		4.62 × 10^−6^/K	
The homogenized mechanical and thermal properties of the upper/lower edge plate flange					
*E*_1_	*E*_2_	*E*_3_	*G*_12_	*G*_13_	*G*_23_
304.5 GPa	304.5 GPa	296.4 GPa	135.38 GPa	135.38 GPa	135.32 GPa
$v_{12}$	$v_{13}$	$v_{23}$	$v_{21}$	$v_{31}$	$v_{32}$
0.149	0.147	0.149	0.152	0.152	0.149
$\lambda_{1}$		$\lambda_{2}$		$\lambda_{3}$	
50.2 W/(m·K)		50.2 W/(m·K)		43.51 W/(m·K)	
$\alpha_{1}$		$\alpha_{2}$		$\alpha_{3}$	
4.7 × 10^−6^/K		4.7 × 10^−6^/K		4.62 × 10^−6^/K	

## Data Availability

The original contributions presented in this study are included in the article. Further inquiries can be directed to the corresponding author.

## References

[1] Okita Y., Mizokami Y., Hasegawa J. (2020). Erosion testing of environmental barrier-coated ceramic matrix composite and its behavior on an aero-engine turbine vane under particle-laden hot gas stream. J. Turbomach..

[2] Ohnabe H., Masaki S., Onozuka M., Kaoru M., Tadashi S. (1999). Potential application of ceramic matrix composites to aero-engine components. Compos. Part A Appl. Sci. Manuf..

[3] Liu X.C., Guo X.J., Xu Y.L., Li L.B., Zhu W., Zeng Y.Q., Li J., Luo X., Hu X.A. (2021). Cyclic thermal shock damage behavior in CVI SiC/SiC high-pressure turbine twin guide vanes. Materials.

[4] Huo S.Y., Yan Q.Y., Gao X., You Y. (2020). Ceramic matrix composite turbine vane thermal simulation test and evaluation. Int. J. Turbo.

[5] Zhang S., Gao X.G., Song Y.D., Wang F., Zhang S.R. (2021). Fatigue behavior and damage evolution of SiC/SiC composites under high-temperature anaerobic cyclic loading. Ceram. Int..

[6] Ruggles-Wrenn M.B., Jones T.P. (2013). Tension–compression fatigue of a SiC/SiC ceramic matrix composite at 1200 ℃ in air and in steam. Int. J. Fatigue.

[7] Ikarashi Y., Ogasawara T., Aoki T. (2019). Effects of cyclic tensile loading on the rupture behavior of orthogonal 3D woven SiC_f_/SiC matrix composites at elevated temperatures in air. J. Eur. Ceram. Soc..

[8] Bhatt R.T., Kalluri S. (2025). Tensile creep and fatigue behaviors of SiC/SiC composites in air under thermal gradient conditions. J. Eur. Ceram. Soc..

[9] Xu Q., Jin X., Liu L., Hou C., Hu N., Chen J., Zhao S., Marrow T.J., Fan X. (2023). Thermal shock and residual strength testing of SiC/SiC composite braided tubes. Exp. Mech..

[10] Kai Q., Xu X.W., Mao C.J., Bui T.Q., Zhang C. (2025). Multiscale damage analysis of through-thickness woven composites under in-plane and out-of-plane loadings: A coupled shell-based model. Compos. Struct..

[11] Liu X., Shen X.L., Gong L.D., Li P. (2018). Multi-scale thermoelastic analysis method for 2D SiC/SiC composite turbine guide vanes. Chin. J. Aeronaut..

[12] Sun Z., Shan Z.D., Shao T.M., Li J.H., Wu X.C. (2021). A multiscale modeling for predicting the thermal expansion behaviors of 3D C/SiC composites considering porosity and fiber volume fraction. Ceram. Int..

[13] Liang S., Wei C., Qiu B.W., Zhang C., Liao H.Y., Ren X.J., Zhang X. (2025). Thermo-mechanical response of SiC_f_/SiC composite cladding: Effect of loss-of-coolant accident duration. Prog. Nucl. Energy.

[14] Zhao X.Y., Guo F., Li B.B., Wang G.N., Ye J.R. (2023). Multiscale numerical modeling for thermal behavior of plain-woven composites with interfacial and internal defects. Int. J. Heat Mass Transf..

[15] Fang G.W., Zheng M.W., Chen M.Z., Gao X.G., Song Y.D. (2024). Stochastic simulation of thermal residual stress in environmental barrier coated 2.5D woven ceramic matrix composites. J. Mater. Eng. Perform..

[16] Singh R.N., Wang H.Y. (1995). Thermal shock behavior of fiber-reinforced ceramic matrix composites. Compos. Eng..

[17] Han J.B., Wang R.Q., Hu D.Y., Liu X., Zhang L., Guo X.J., Cho C.D. (2022). Multi-scale analysis and experimental research for turbine guide vanes made of 2D braided SiC_f_/SiC composites in high-cycle fatigue regime. Int. J. Fatigue.

[18] Reynaud P., Dalmaz A., Tallaron D., Rouby D., Fantozzi G. (1993). Fatigue behaviour related to interface modification during load cycling in ceramic-matrix fibre composites. Compos. Sci. Technol..

[19] Wang S.F., Zhang Z. (2020). Failure mechanism of turbine guide vane and oxide composition analysis on the surface of failure vane cracks. Eng. Fail. Anal..

[20] Liu Y., Qu Z., Guo J., Zhao X.M. (2019). Numerical study on effective thermal conductivities of plain woven C/SiC composites with considering pores in interlaced woven yarns. Int. J. Heat Mass Tran..

[21] Dong K., Liu K., Zhang Q., Gu B.H., Sun B.Z. (2016). Experimental and numerical analyses on the thermal conductive behaviors of carbon fiber/epoxy plain woven composites. Int. J. Heat Mass Tran..

[22] Xu Y., Ren S., Zhang W. (2018). Thermal conductivities of plain woven C/SiC composite: Micromechanical model considering PyC interphase thermal conductance and manufacture-induced voids. Compos. Struct..

[23] Sun Z., Shan Z., Huang H., Wang D., Wang W., Liu J., Tan C., Chen C. (2024). Multi-scale Modeling and Finite Element Analyses of Thermal Conductivity of 3D C/SiC Composites Fabricating by Flexible-Oriented Woven Process. Chin. J. Mech. Eng..

[24] Dutra T.A., Ferreira R.T.L., Resende H.B., Guimaraes A., Guedes J.M. (2020). A complete implementation methodology for asymptotic homogenized using a finite element commercial software: Preprocessing and postprocessing. Compos. Struct..

[25] Christoff B.G., Brito-Santana H., Talreja R., Tita V. (2020). Development of an abaqus™ plug-in to evaluate the fourth-order elasticity tensor of a periodic material via homogenization by the asymptotic expansion method. Finite Elem. Anal. Des..

[26] (2015). Test Method for Tensile Properties of Continuous Fiber—Reinforced Ceramic Composites at Ambient Temperature.

[27] (2008). Determination of Thermal Diffusivity or Thermal Conductivity by the Flash Method.

[28] (2008). Fine Ceramics (Advanced Ceramics, Advanced Technical Ceramics)—Test Method for Linear Thermal Expansion of Monolithic Ceramics by Push-Rod Technique.

[29] (2010). Test Methods for Density and Apparent Porosity of Fine Ceramics.

[30] He Z.P., Zhang R.Q., Fu D.G., Li M., Chen Z.K., Qiu S.Y. (2019). Tensile mechanical behavior of SiC fiber bundle reinforced composites with different interfaces. J. Mater. Eng..

[31] Zhang X.D., Deng J.K., Ma D.Y., Cao H.Y., Zhang R.Q., Tang R. (2022). Effective thermal conductivity and microstructure design of fully ceramic microencapsulated fuel pellet based on finite element calculations. J. Lanzhou Univ. Technol..

[32] Dong B., Yu C., Deng C.J., Zhu H.X., Ding J., Tang H. (2023). Research progress in thermal conductivity of SiC ceramics. J. Mater. Eng..

[33] Li D.Y., Han F., Wang J.X., Xu P., Zhong Z.X. (2019). Effect of pore-forming agent carbon powder on properties of SiC membrane supports. Membr. Sci. Technol..

[34] Kiradjiev K.B., Halvorsen S.A., Gorder R.A.V., Howison S.D. (2019). Maxwell-type models for the effective thermal conductivity of a porous material with radiative transfer in the voids. Int. J. Therm. Sci..

[35] Anurag J., Burri A.C., Sandhyarani B. (2023). Modified Halpin–Tsai model for predicting the mechanical properties of polymer composites reinforced with different cross-sectional fibers. Mater. Today.

[36] Wu Y.P., Jia Q.X., Yu D.S., Zhang L.Q. (2004). Modeling Young’s modulus of rubber–clay nanocomposites using composite theories. Polym. Test..

[37] Kim H.S., Hong S.I., Kim S.J. (2021). On the rule of mixtures for predicting the mechanical properties of composites with homogeneously distributed soft and hard particles. J. Mater. Process. Technol..

[38] Shen X.L., Zhang S., Liu X., Gong L.D., Dong S.J. (2021). Prediction of the thermo-mechanical properties of the SiC_f_/SiC RVE model via FEM and asymptotic homogenization method: Process and implementation details. Arch. Comput. Method Eng..

[39] Liu H.X., You C., Wu Q.Y., Gao X.G., Song Y.D. (2025). Thermal stress analysis of CMC turbine guide vane considering thermal-mechanical coupling effect. J. Propul. Technol..

[40] Ramos G.R., Dos-Santos T., Rossi R. (2017). An extension of the hill-mandel principle for transient heat conduction in heterogeneous media with heat generation incorporating finite RVE thermal inertia effects. Int. J. Numer. Methods Eng..

[41] Ni Z., Yu G.Q., Chen Y.C., Xue B.C., Deng Y.F., Ma W.B., Gao X.G., Song Y.D. (2024). Experimental and numerical analysis of CMCs mechanical properties under high-temperature thermal gradient environment. Ceram. Int..

[42] Ding S., Zeng Y., Jiang D. (2006). Thermal shock resistance of in situ reaction bonded porous silicon carbide ceramics. Mater. Sci. Eng. A.

[43] Zhang Y.J., Obara E., Wang S., Zhu L.Y., Li W.D., Lin S.Y., Han Z.L., Luo C.Y. (2025). High-temperature tensile failure mechanism of RTM-made composite T-joints. Def. Technol..

